# Supplementary material for: Short-termed changes in quantitative ultrasound estimated bone density among young men in an 18-weeks follow-up during their basic training for the Swiss Armed Forces
Source: PeerJ. 2023 Apr 6;11:e15205. doi: 10.7717/peerj.15205 (PMC10083003; doi:10.7717/peerj.15205)
Supplement: Supplemental Information 1 [file peerj-11-15205-s001.docx]

**APPENDIX MATERIAL**

**Appendix Tables**

**Appendix Table 1.** Descriptive characteristics of subjects at start and 18-weeks later at follow-up (n=73), subdivided into groups “sports” and “non-sports”. Only statistically significant results are shown. Subjects who could not be reassessed in follow-up were excluded from the study (n=31) and aren’t in this table. SD=standard deviation. p-value with level of significance < 5 %. BUA=bone ultrasound attenuation. MET=metabolic equivalents per week.

| **Groups** | **Characteristics** | **start** | **±** | **SD** | **follow-up** | **±** | **SD** | **p-Value** |  |
| --- | --- | --- | --- | --- | --- | --- | --- | --- | --- |
| **Sport** | |  |  |  |  |  |  |  |  |
|  | Bone measurements |  |  |  |  |  |  |  |  |
|  | BUA (dB/MHz) | 93.4 | ± | 16.1 | 85.6 | ± | 15.5 | < 0.001 | *** |
|  | Anthropometrics |  |  |  |  |  |  |  |  |
|  | Gripstrength (kg) | 47.5 | ± | 9.1 | 52.8 | ± | 8.7 | < 0.001 | *** |
|  | Fat mass (%) | 13.5 | ± | 6.0 | 14.7 | ± | 5.8 | 0.006 | ** |
|  | Weight (kg) | 73.9 | ± | 11.0 | 74.8 | ± | 10.7 | 0.045 | * |
|  | Height (m) | 1.789 | ± | 0.1 | 1.793 | ± | 0.1 | < 0.001 | *** |
|  | Physical activity (MET) | 7140.9 | ± | 6115.7 | 4194.4 | ± | 4025.4 | 0.006 | ** |
| **Non-sport** | |  |  |  |  |  |  |  |  |
|  | Bone measurements |  |  |  |  |  |  |  |  |
|  | BUA (dB/MHz) | 79.7 | ± | 14.7 | 74.9 | ± | 15.0 | 0.011 | * |
|  | Anthropometrics |  |  |  |  |  |  |  |  |
|  | Gripstrength (kg) | 48.2 | ± | 10.2 | 50.6 | ± | 9.8 | 0.016 | * |
|  | Physical activity (MET) | 8131.3 | ± | 7240.2 | 3272.0 | ± | 2851.2 | < 0.001 | *** |

**Appendix Table 2.** Descriptive characteristics of subjects at start and 18-weeks later at follow-up (n=73), subdivided into smoker groups. Only statistically significant results are shown. Subjects who could not be reassessed in follow-up were excluded from the study (n=31) and aren’t in this table. SD=standard deviation. p-value with level of significance < 5 %. BUA=bone ultrasound attenuation. MET=metabolic equivalents per week.

| **Groups** | **Characteristics** | **start** | **±** | **SD** | **follow-up** | **±** | **SD** | **p-Value** |  |
| --- | --- | --- | --- | --- | --- | --- | --- | --- | --- |
| **Smoker** | |  |  |  |  |  |  |  |  |
|  | Bone measurements |  |  |  |  |  |  |  |  |
|  | BUA (dB/MHz) | 85.4 | ± | 16.9 | 78.9 | ± | 17.3 | < 0.001 | *** |
|  | Anthropometrics |  |  |  |  |  |  |  |  |
|  | Gripstrength (kg) | 49.3 | ± | 10.4 | 53.4 | ± | 9.0 | < 0.001 | *** |
|  | Fat mass (%) | 14.0 | ± | 8.3 | 15.1 | ± | 8.1 | 0.018 | * |
|  | Height (m) | 1.78 | ± | 0.1 | 1.79 | ± | 0.1 | 0.016 | * |
|  | Physical activity (MET) | 8848.8 | ± | 6778.4 | 4037.6 | ± | 3381.1 | < 0.001 | *** |
| **Non-smoker** | |  |  |  |  |  |  |  |  |
|  | Bone measurements |  |  |  |  |  |  |  |  |
|  | BUA (dB/MHz) | 89.9 | ± | 16.7 | 83.2 | ± | 14.9 | 0.002 | ** |
|  | Anthropometrics |  |  |  |  |  |  |  |  |
|  | Gripstrength (kg) | 46.5 | ± | 8.5 | 50.6 | ± | 9.2 | < 0.001 | *** |
|  | Weight (kg) | 71.6 | ± | 10.6 | 72.4 | ± | 10.5 | 0.019 | * |
|  | Height (m) | 1.781 | ± | 0.1 | 1.784 | ± | 0.1 | 0.005 | ** |
|  | Physical activity (MET) | 6413.8 | ± | 6250.2 | 3621.5 | ± | 3807.8 | 0.007 | ** |

**Appendix Figures**

**Appendix Figure 1A-D.** Changes of variables regarding to starting point. These figures illustrate the regression-to-the-mean-like behavior of variables during the basic training (delta: second measurement – first measurement) in association with the starting point (first measurement). Figure 1A physical activity: Subjects with high physical activity before start the basic training (first measurement) nearly lost all of their high activity level. Same behavior of variables are found in Physical activity (Appendix Figure 1B), Skeletal muscle mass (Appendix Figure 1C), Visceral adipose tissue (Appendix Figure 1D), Speed of Sound (Appendix Figure 1E) and Bone Ultrasound Attenuation (Appendix Figure 1F). R=Pearson correlation coefficient. p-value with level of significance < 5 %. SOS=speed of sound. BUA=bone ultrasound attenuation. MET=metabolic equivalents per week. Red line=regression line.

**Appendix Figure 2A-D.** Associations of variables with SOS. These figures illustrate changes of variables during the basic training (delta: second measurement – first measurement) in association with the changes of bone parameter SOS. Appendix Figure 2A: Weakly positive association between grip strength and SOS. Subjects with higher grip strength (delta) had also higher SOS (delta) after the basic training. Appendix Figure 2B: Weakly negative correlation between fat free mass and SOS. Subjects, who gained fat free mass lost SOS during basic training. Appendix Figure 2C: Weakly negative correlation between skeletal muscle mass and SOS. Subjects, who gained skeletal muscle mass lost SOS during basic training. Appendix Figure 2D: Slight negative correlation between waist circumference and SOS. Subjects with higher waist circumference had lower SOS after the basic training. R=Pearson correlation coefficient. p-value with level of significance < 5 %. SOS=speed of sound. Red line=regression line.

**Appendix Figure 3A-B.** Associations of variables with BUA. Appendix Figure 3A: Slightly negative correlation between change of physical activity and change of BUA. The more subjects lost physical activity, the higher BUA resulted after the basic training. Appendix Figure 3B: Weakly negative correlation between relative fat mass and BUA. Gaining fat mass is associated with lower BUA after the basic training. R=Pearson correlation coefficient. p -value with level of significance < 5 %. BUA=bone ultrasound attenuation. MET=metabolic equivalents per week. Red line=regression line.

**Appendix Figure 4.** Association of physical activity before basic training with change of BUA. This figure illustrates change of BUA during the basic training (delta: second measurement – first measurement) in association with the physical activity before start the basic training (first measurement). Subjects with high activity before start the basic training had higher BUA after 18-weeks. R=Pearson correlation coefficient. p-value with level of significance < 5 %. BUA=bone ultrasound attenuation. MET=metabolic equivalents per week. Red line=regression line.
